# Evolutionary Dynamics of GLD-1–mRNA Complexes in *Caenorhabditis* Nematodes

**DOI:** 10.1093/gbe/evu272

**Published:** 2014-12-09

**Authors:** Alana V. Beadell, Eric S. Haag

**Affiliations:** ^1^Program in Behavior, Evolution, Ecology, and Systematics, University of Maryland, College Park; ^2^Department of Biology, University of Maryland, College Park; ^3^Present address: Department of Organismal Biology and Anatomy, University of Chicago, Chicago, IL

**Keywords:** RNA-binding proteins, STAR family, translational control, molecular evolution

## Abstract

Given the large number of RNA-binding proteins and regulatory RNAs within genomes, posttranscriptional regulation may be an underappreciated aspect of *cis*-regulatory evolution. Here, we focus on nematode germ cells, which are known to rely heavily upon translational control to regulate meiosis and gametogenesis. GLD-1 belongs to the STAR-domain family of RNA-binding proteins, conserved throughout eukaryotes, and functions in *Caenorhabditis elegans* as a germline-specific translational repressor. A phylogenetic analysis across opisthokonts shows that GLD-1 is most closely related to *Drosophila* How and deuterostome Quaking, both implicated in alternative splicing. We identify messenger RNAs associated with *C. briggsae* GLD-1 on a genome-wide scale and provide evidence that many participate in aspects of germline development. By comparing our results with published *C. elegans* GLD-1 targets, we detect nearly 100 that are conserved between the two species. We also detected several hundred Cbr-GLD-1 targets whose homologs have not been reported to be associated with *C. elegans* GLD-1 in either of two independent studies. Low expression in *C. elegans* may explain the failure to detect most of them, but a highly expressed subset are strong candidates for Cbr-GLD-1-specific targets. We examine GLD-1-binding motifs among targets conserved in *C. elegans* and *C. briggsae* and find that most, but not all, display evidence of shared ancestral binding sites. Our work illustrates both the conservative and the dynamic character of evolution at the posttranslational level of gene regulation, even between congeners.

## Introduction

Although many studies have investigated the nature of *cis* and *trans* changes in transcription factors and their binding sites across species (e.g., Bradley et al. 2010; McDaniell et al. 2010; Schmidt et al. 2010; Ni et al. 2012), the contribution that changes in RNA-binding proteins (RBPs) and their targets may make to evolution has been studied little. The STAR-domain (for *s*ignal *t*ransduction and *a*ctivation of *R*NA metabolism) family of RBPs is found throughout eukaryotes. Work in *Drosophila*, mice, *Xenopus*, *Caenorhabditis*, and human cell lines has found that STAR-domain proteins participate in a range of molecular processes in both the nucleus and the cytoplasm, including RNA translational repression, alternative splicing, and nuclear export. These, in turn, are essential for biological processes such as cell division and cell differentiation in early and late development (e.g., Kramer and Utans 1991; Francis, Barton, et al. 1995; Francis, Maine, et al. 1995; Baehrecke 1997; Zorn and Krieg 1997; Li et al. 2000; Nabel-Rosen et al. 2005; Paronetto et al. 2009; Fu et al. 2012; Monk et al. 2010; Klein et al. 2013). STAR-domain proteins have also been linked to human pathologies, such as cancer, spinal muscular atrophy, obesity, fragile X tremor/ataxia syndrome, and infertility (Pedrotti et al. 2010; Richard 2010; Sellier et al. 2010; Chen et al. 2012; Huot et al. 2012; Klein et al. 2013) and in the evolutionary divergence between species (Beadell et al. 2011).

Signal transduction and activation of RNA metabolism (STAR)-domain proteins are characterized by a single K-homology (KH) domain flanked by two domains named Qua1 and Qua2 after the homolog Quaking in mice (Ebersole et al. 1996). Qua1 mediates the homodimerization of STAR-domain proteins, except within the SF1 (splicing factor 1) subfamily that remains as monomers (Zorn and Krieg 1997; Liu et al. 2001; Ryder et al. 2004; Beuck et al. 2010; Meyer et al. 2010; Teplova et al. 2013). The KH and the Qua2 domains provide an extended RNA-binding surface (Liu et al. 2001; Ryder et al. 2004; Maguire et al. 2005; Teplova et al. 2013). STAR-domain proteins form contacts with different protein-binding partners and are themselves controlled by translational modifications (e.g., Resnick et al. 1997; Di Fruscio et al. 1999; Clifford et al. 2000; Stoss et al. 2001; Côté et al. 2003; Selenko et al. 2003; Zhang et al. 2003; Robard et al. 2006; Huot et al. 2009; Nir et al. 2012).

STAR-domain proteins exert their effects by binding to and influencing the fate of RNA molecules. Early work identified some of these RNA targets on an individual or small scale (e.g., Lee and Schedl 2001; Itoh et al. 2002; Matter et al. 2002; Nabel-Rosen et al. 2002; Wu et al. 2002; Di Fruscio et al. 2003; Mootz et al. 2004; Tremblay and Richard 2006; Israeli et al. 2007). More recently, methods such as splicing-sensitive microarrays, in vitro protein–RNA binding assays, and in vitro nucleic acid selection followed by in silico genome searches (Ryder et al. 2004; Galarneau and Richard 2005; Galarneau and Richard 2009) as well as genome-wide approaches such as RNA immunoprecipitation (RIP)-chip (Wright et al. 2011) and photoreactive ribonucleoside-crosslinking/immunoprecipitation (PAR-CLIP) (Hafner et al. 2010; Jungkamp et al. 2011) have been employed to identify the RNA recognition sites and targets of STAR-domain proteins.

GLD-1 (defective in germline development 1) is a cytoplasmic and germline-specific STAR-domain translational repressor of messenger RNAs (mRNAs) in *Caenorhabditis elegans,* and functions in the mitosis/meiosis decision of germline cells, meiotic progression of oocyte-fated cells, and hermaphrodite sperm production (Goodwin et al. 1993; Francis, Barton, et al. 1995; Francis, Maine, et al. 1995; Jones et al. 1996; Jan et al. 1999; Lee and Schedl 2001). The GLD-1 orthologs of *C. elegans* and its congener *C. briggsae* have high sequence identity, have similar temporal and spatial protein expression patterns, and repress the translation of at least one common mRNA target, the yolk receptor *rme-2* (Nayak et al. 2005). In both species, GLD-1 is important for germline development, and *C. briggsae* GLD-1 coding plus regulatory sequences can rescue *C. elegans* hermaphrodites lacking endogenous *gld-1* function (Beadell et al. 2011). Together, these findings suggest that GLD-1 has maintained similar biological roles and biochemical activity over roughly 20 Myr of evolutionary divergence between *C. elegans* and *C. briggsae* (Cutter 2008). Nevertheless, GLD-1 has been independently coopted to regulate germline sex in these convergently hermaphroditic species, and its roles in this are opposite: Cel-GLD-1 promotes sperm production by negatively regulating *tra-2* translation, whereas Cbr-GLD-1 promotes oocyte development, in part by interacting with *puf-8* (Goodwin et al. 1993; Nayak et al. 2005; Beadell et al. 2011). The contextual differences responsible for this appear to include both species-specific protein cofactors (Nayak et al. 2005) and alterations of target mRNA sequences and their own functions downstream of GLD-1 regulation (Beadell et al. 2011). To examine the evolution of GLD-1 action more fully, we identified the mRNA targets of endogenous *C. briggsae* GLD-1 on a genome-wide scale using immunoprecipitation (IP) and microarrays (RIP-chip; Tenenbaum et al. 2000). Comparisons with previously published studies of *C. elegans* GLD-1 targets reveal both conserved and dynamic targets. Our findings support the idea that although the GLD-1 RBP has remained stable over many tens of millions of years of nematode evolution, its RNA targets are not similarly static.

## Results

### Evolutionary History of STAR-Domain Proteins

**Fig. 1.—:**
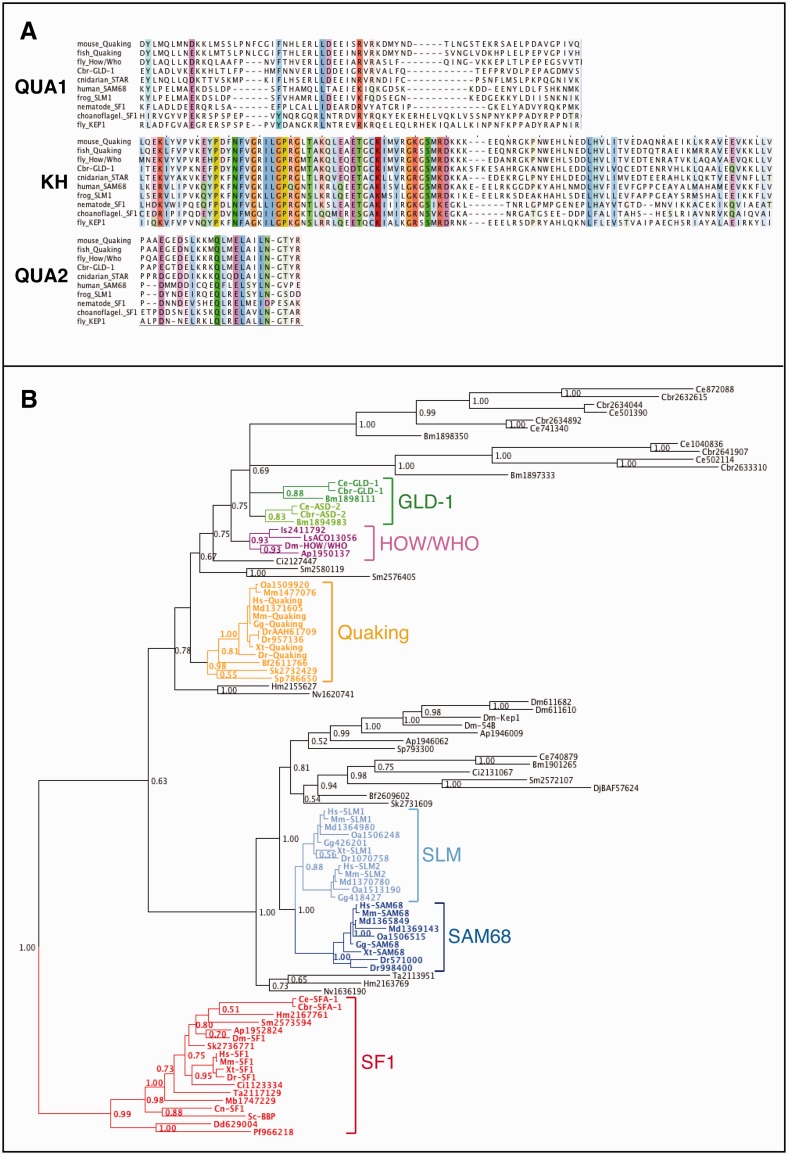
Conservation and evolutionary history of GLD-1. (*A*) Alignment of the three regions that define the STAR-domain protein family from representative members. Residues are colored using a 33% similarity threshold and highlight chemically similar amino acids. (*B*) Bayesian phylogenetic tree of the STAR-domain protein family in representative opisthokonts plus two outgroups. The tree is rooted at the SF1 clade of ancient splicing factors. Node posterior probabilities are given. Subfamilies of well-studied STAR proteins are highlighted: GLD-1 and ASD-2 in dark green and light green, respectively; How/Who in purple; Quaking in orange; SAM68 (KHDRBS1) and SLM-1/SLM-2 (KHDRBS2/KHDRBS3, respectively) in dark blue and light blue, respectively; and SF1 in red. Proteins are named with their genus/species abbreviation followed by either their protein name (e.g., “Quaking”) or their NCBI “NP_” or “XP_” accession number. Mb, *Monosiga brevicollis* (choanoflagellate), Ta, *Trichoplax adhaerens* (placazoan), Nv, *Nematostella vectensis* (cnidarian), Hm, *Hydra magnipapillata* (cnidarian), Bm, *Brugia malayi* (nematode), Cbr, *Caenorhabditis briggsae* (nematode), Ce, *Caenorhabditis elegans* (nematode), Ap, *Acyrthosiphon pisum* (arthropod), Is, *Ixodes scapularis* (arthropod), Dm, *Drosophila melanogaster* (arthropod), Ls, *Lepeophtheirus salmonis* (arthropod), Sm, *Schistosoma mansoni* (Platyhelminthes), Dj, *Dugesia japonica* (Platyhelminthes), Sp, *Strongylocentrotus purpuratus* (echinoderm), Sk, *Saccoglossus kowalevskii* (hemichordate), Bf, *Branchiostoma floridae* (chordate), Ci, *Ciona intestinalis* (chordate), Dr, *Danio rerio* (chordate), Xt, *Xenopus tropicalis* (chordate), Gg, *Gallus gallus* (chordate), Oa, *Ornithorhynchus anatinus* (chordate), Md, *Monodelphis domestica* (chordate), Mm, *Mus musculus* (chordate), Hs, *Homo sapiens* (chordate), Sc, *Saccharomyces cerevisiae* (Ascomycota), Cn, *Cryptococcus neoformans* (Basidiomycota), Dd, *Dictyostelium discoideum* (Ameobozoa), Pf, *Plasmodium falciparum* (Apicomplexa).

Before examining the evolution of RBP targets, it is essential that orthologous proteins are being compared. To better understand the relationships of STAR-domain proteins, we used the well-conserved STAR domain (fig. 1*A*) to identify related sequences across the opisthokonts (the eukaryote clade that includes animals and fungi). In total, 96 homologs from representative taxa were identified, and their relationships resolved using Bayesian inference, rooted with the SF1 clade of ancient splicing factors (Collins and Penny 2005) (fig. 1*B*). The resulting phylogeny demonstrates that GLD-1 belongs to a large, nematode-specific STAR subfamily, some members of which are unusually divergent. This subfamily is itself nested within a larger clade containing the arthropod How/Who and the deuterostome Quaking proteins. Single proteins from the cnidarians, *Nematostella* and *Hydra*, lie at the base of the GLD-1/HOW/Quaking clade. Because all major groups of metazoans possess a GLD-1/How/Quaking subfamily homolog, we infer that the cnidarian–bilaterian ancestor possessed a GLD-1/How/Quaking-like protein.

Vertebrate SAM68 and SLM-like/KHDRBS proteins also form a monophyletic group, falling within a deep clade that contains the *Drosophila* protein Kep1, involved in alternative splicing and oogenesis (Di Fruscio et al. 2003; Robard et al. 2006). Just as for the GLD-1/How/Quaking clade described above, the cnidarian proteins are basal within this clade, indicating that the metazoan ancestor also possessed a SAM68/SLM-like (KHDRBS) protein.

The SF1 subfamily of STAR-domain proteins is involved in branch point recognition during mRNA splicing. The SF1-containing clade possesses representatives from all taxa in the tree, including the sole sequences recovered from fungi, choanoflagellates (the sister taxon to metazoans), and the two nonopisthokont outgroups, *Dictyostelium* and *Plasmodium*. This topology is consistent with SF1’s role in an ancestral process of mRNA splicing. Our analysis suggests that the ancestral opisthokont had a single SF1-like homolog and that animals later evolved both GLD-1/HOW/Quaking-like and SAM68-like genes by duplication before their radiation.

### Identification of *C. briggsae* GLD-1-Associated mRNAs

**Fig. 2.—:**
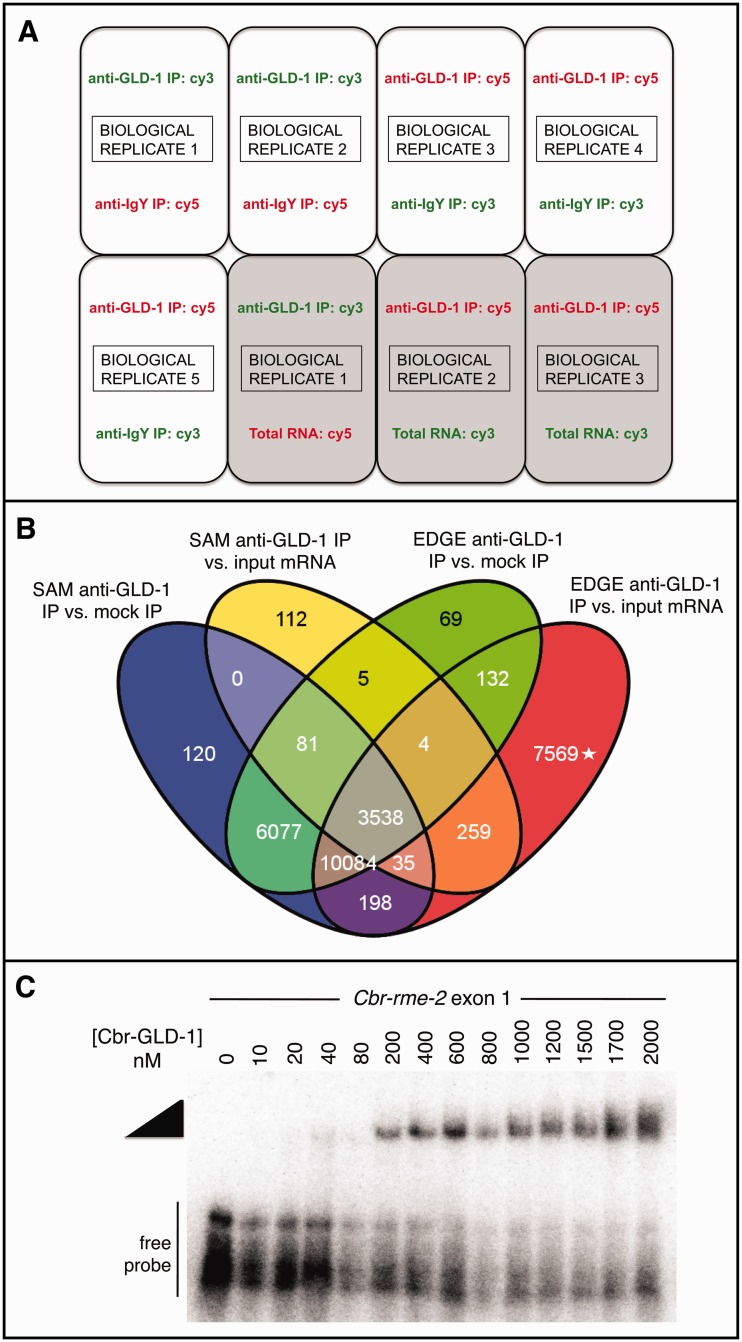
Mircoarray experimental design and analysis to identify Cbr-GLD-1-associated RNAs. (*A*) Schematic of microarray design for two different expression comparisons, anti-GLD-1 IP mRNA versus mock anti-IgY IP mRNA and anti-GLD-1 IP mRNA versus total input mRNA. Dye swaps and biological replicates are incorporated. (*B*) Overlap of positive probes from the two microarray comparisons, anti-GLD-1 IP mRNA versus mock anti-IgY IP mRNA and anti-GLD-1 IP mRNA versus total input mRNA, each analyzed with two differential gene expression programs, SAM and EDGE. Values in each oval are the number of probes enriched in anti-GLD-1 IPs with FDRs of at most less than 2%. (The group of probes marked with the asterisk is enriched in both anti-GLD-1 IPs and total input mRNA.) In total, 3,538 probes were found enriched in common to all four data sets, representing 965 different *Caenorhabditis briggsae* protein coding genes. (*C*) The STAR domain of Cbr-GLD-1 can shift an RNA fragment of exon 1 RIP-chip target *Cbr-rme-2*, which contains multiple sequence motifs consistent with GLD-1 binding, in a concentration-dependent manner. Wedge, Cbr-GLD-1-dependent protein–RNA complex. A negative control RNA fragment from the 5′-UTR of *Cbr-tra-2* does not shift at these concentrations (data not shown).

To compare the target mRNAs of *C. briggsae* and *C. elegans* GLD-1 orthologs, we immunoprecipitated endogenous Cbr-GLD-1-associated mRNAs from hermaphrodites using an antibody that recognizes Cbr-GLD-1 protein in native form in whole tissues (supplementary fig. S1, Supplementary Material online). We then used custom *C. briggsae* microarrays to identify Cbr-GLD-1-associated transcripts. This differs from a typical microarray experiment in that it is not a straightforward comparison of transcriptome-wide expression. We therefore employed two different comparisons to eliminate likely artifacts. First, we compared mRNA from anti-Cbr-GLD-1 IP with those from a control anti-IgY mock IP. The goal here was to eliminate transcripts that nonspecifically adhere to the bead-immunoglobulin complexes. However, because GLD-1 is an RBP, it may reproducibly bind nontargets with low affinity during lysate formation. We therefore further compared the anti-Cbr-GLD-1IP RNA with unmanipulated total input mRNA, and required that Cbr-GLD-1-associated transcripts be enriched beyond the extent predicted by their abundance (fig. 2*A*).

RIP-chip violates important assumptions of many microarray analysis methods. For example, it is common to treat intensity-dependent biases across treatments as artifacts in standard microarray processing methods, but here, the intensity signals from true *C. briggsae* mRNA targets should increase as the abundance of those mRNAs increases, whereas the signals for those same probes in the mock IP channels should remain at baseline levels (supplementary fig. S2, Supplementary Material online). Further, as most *C. briggsae* mRNAs are not bound by GLD-1, the microarray probes for most gene products will have near-zero fluorescent measurements, which violates the assumption that most genes between treatments and controls are not differentially expressed and are also normally distributed (supplementary fig. S3, Supplementary Material online). The intensity measurement distributions resulting from the RIP-chip procedure are not tolerated by standard microarray processing and gene expression comparison methods (Cui et al. 2003; Grant et al. 2007; Russell et al. 2009).

To account for the nonnormality and intensity-dependent bias of our data, we evaluated different microarray processing methods in a systematic fashion (supplementary fig. S3, Supplementary Material online). To address possible corrections for background intensity differences within arrays, we compared no background subtraction (Cui et al. 2003; Zahurak et al. 2007; Russell et al. 2009) to the *normexp* function implemented in *limma* (Wettenhall and Smyth 2004; Ritchie et al. 2007; Silver et al. 2009). We also tested two normalization methods that control for technical differences in intensity measurements between arrays and also between fluorescent dyes: Median scaling and eCADS (Grant et al. 2007; Dabney and Storey 2007). The most uniform box plots across arrays for the anti-GLD-1 IP versus anti-IgY IP mRNA expression comparison resulted from *normexp* background-correction and eCADS normalization, whereas the most uniform box plots for the anti-GLD-1 IP versus total mRNA expression comparison resulted from combining no background correction with eCADS normalization (supplementary fig. S3, Supplementary Material online).

To detect statistically significant intensity differences among probes in both comparisons, and to reduce detection bias inherent in any one analysis method, we employed two nonparametric analysis programs, SAM (Tusher et al. 2001) and EDGE (Leek et al. 2006). To conservatively identify GLD-1-associated mRNAs, we retained only probes in common to both anti-GLD-1 IP versus anti-IgY IP and anti-GLD-1 IP versus total input mRNA comparisons at a false discovery rate (FDR) of no greater than 2% for any particular comparison. This resulted in 3,538 probes called significant by both differential expression detection programs (fig. 2*B*), corresponding to 965 *C. briggsae* protein-coding genes (supplementary table S2, Supplementary Material online). We also confirmed Cbr-GLD-1 association for several likely-to-be conserved targets by quantitative reverse transcriptase polymerase chain reaction (PCR) (Beadell et al. 2011; Materials and Methods), and direct association of a previously identified target, *Cbr-rme-2* (Nayak et al. 2005) using recombinant Cbr-GLD-1 protein (fig. 1*C*).

### Knockdown Phenotypes of Putative *C. briggsae* GLD-1 Targets

Having identified a likely set of mRNAs that are bound by *C. briggsae* GLD-1, we next sought to determine whether they function in the germ line, as expected. *Caenorhabditis elegans* GLD-1 is germline-specific, and acts in stem cell mitosis, oogenesis, cell fate commitment, sex determination, and is present in the early embryo (Francis, Barton et al. 1995; Francis, Maine, et al. 1995; Jones et al. 1996; Jan et al. 1999). Given the similar expression pattern of GLD-1 in *C. briggsae* and *C. elegans* and what is known about *C. briggsae* GLD-1’s biological roles in the germ line (Nayak et al. 2005; Beadell et al. 2011; Liu et al. 2012), we expected that RNAi knockdown of true GLD-1 targets would primarily produce defects in germline development or embryogenesis. In total, 125 of the GLD-1-associated mRNA lists (13%) were selected for knockdown. Their probes were among the highest scoring either by SAM statistic or by fold-change calculation in either the mock IP or total input mRNA comparisons (supplementary fig. S4, Supplementary Material online). We first injected pairs of double-stranded RNA (Gonczy et al. 2000) into adult *C. briggsae* hermaphrodites and analyzed their self-progeny. Follow-up analyses were conducted using single-gene injections.

Table 1 lists the RNAi phenotypes observed. The most common knockdown phenotype was defective oogenesis, where oocytes were small and/or unusually shaped and/or had an unusual appearance (e.g., “dimpling”). In a few cases, we found what looked to be disintegration of the most proximal oocyte(s). In most germ lines with morphologically aberrant oocytes, we observed sperm clumped in the uterus, not localized to the spermathecae (data not shown). Other germline phenotypes observed include reduced germ cell number; meiotic arrest of germ cells and/or delayed gametogenesis; slow embryo laying (perhaps due to slow ovulation, fertilization, and/or embryo extrusion); the laying of unfertilized, shell-less oocytes despite the presence of sperm; decaying germ cells (either in the proximal and/or distal germline); and slow growth and/or sickness of F1 larvae. Control RNAi injections directed against GFP did not produce germline phenotypes (not shown). Overall, 34 of 65 (52.3%) pairs of putative *C. briggsae* GLD-1 targets and 14 of 25 (56%) singly injected targets produced obvious germline RNAi knockdown phenotypes. This frequency is comparable to that observed in a similar injection-based screen of known germline genes in *C. elegans* (Colaiacovo et al. 2002), and much greater than observed in whole-genome knockdown studies (e.g., Simmer et al. 2003).

**Table 1:** RNA Interference Knockdown Phenotypes of Putative *Caenorhabditis briggsae* GLD-1 Target mRNAs

(*A*) Paired RNAi Experiments			
Pair	dsRNA 1	dsRNA 2	Knockdown Phenotype(s) Observed
1	CBG02483	CBG09734	Pvl; few germ cells; gametes malformed
2	*Cbr-pie-1*	CBG03777	Low brood size from hermaphrodites with a normal-looking germline
3	*Cbr-spn-4*	*Cbr-set-14*	
4	*Cbr-skr-1*	*Cbr-hop-1*	
5	*Cbr-pos-1*	CBG07193	
6	CBG09898	*Cbr-daz-1*	Low brood size from hermaphrodites with a normal-looking germline
7	*Cbr-pal-1*	*Cbr-rme-2*	Aberrant oocytes
8	*Cbr-oma*	*Cbr-tag-246*	Misshapen uterine tissue; aberrant oocytes; few embryos
9	*Cbr-arl-8*	*Cbr-swd-3.3*	
10	*Cbr-patr-1*	*Cbr-rskn-1*	
11	*Cbr-puf-6.1*	*Cbr-puf-8*	Few germ cells
12	CBG22317	*Cbr-nos-2*	
13	CBG08921	CBG04207	Slow growth of progeny
14	CBG05292	CBG01393	
15	CBG16726	CBG05879	
16	CBG08527	CBG11569	
17	CBG05095	CBG09653	
18	CBG10091	CBG13227	
19	CBG04372	CBG04373	
20	CBG03080	CBG09925	
21	CBG20875	CBG14962	
22	*Cbr-egg-4*	*Cbr-tpa-1*	Injected worms lay only shell-less oocytes
23	CBG00199	CBG22683	
24	CBG01956	CBG02251	Low brood size from hermaphrodites with a normal-looking germline
25	CBG0282R	CBG03076	Aberrant oocytes; “disintegrating” proximal oocytes
26	CBG03085	CBG03615	No germ cells
27	CBG04301	CBG04302	Aberrant oocytes
28	CBG21596	CBG20384	Aberrant oocytes
29	CBG14085	*Cbr-aly-1*	Aberrant oocytes
30	CBG13508	CBG07045	Aberrant oocytes
31	CBG07640	CBG07661	Aberrant oocytes, “disintegration” of most proximal oocyte
32	CBG08571	CBG08989	Aberrant oocytes
33	CBG09062	CBG09108	Delayed gametogenesis
34	CBG09250	CBG09264	Aberrant oocytes
35	CBG09348	CBG09840	Few germ cells; aberrant oocytes
36	CBG10477	CBG10809	Aberrant proximal oocytes and postpachytene cells
37	CBG11199	*Cbr-mes-3*	
38	CBG20654	*Cbr-moe-3*	Slow-growing, Sma, Egl, Pvl; abnormal somatic gonad migration, aberrant oocytes
39	CBG12306	CBG07050	Delayed gametogenesis, aberrant oocytes, abnormal embryonic cell divisions
40	*Cbr-puf-3.1*	*Cbr-puf-3.2*	
41	CBG13131	*Cbr-mop-25.3*	Aberrant oocytes; lay oocytes instead of embryos
42	*Cbr-lir-1*	CBG03256	Low brood size from hermaphrodites with a normal-looking germline
43	CBG06213	*Cbr-mex-3*	100% Let
44	*Cbr-glp-1*	*Cbr-nhr-43*	100% Let
45	CBG05635	CBG05978	
46	CBG02511	CBG02683	Aberrant oocytes; lay oocytes instead of embryos
47	*Cbr-dmd-6*	*Cbr-unc-71*	
48	CBG01946	CBG09113	Low penetrance necrosis throughout germline
49	CBG04364	CBG11013	Necrosis of distal and proximal germline, aberrant oocytes
50	CBG05916	CBG11273	Aberrant oocytes and some “disintegrating” oocytes
51	*Cbr-alg-2*	CBG14486	
52	CBG12096	CBG20835	Ste; no stacking oocytes, but also few visible sperm
53	CBG11806	CBG24315	
54	CBG12313	CBG19423	
55	CBG13382	CBG19713	
56	CBG13621	CBG23246	
57	CBG13812	CBG14565	
58	CBG14133	CBG14242	Aberrant oocytes, some germ cells' sex not discernable
59	*Cbr-skr-16*	CBG16225	
60	CBG14796	CBG15553	
61	CBG16460	CBG22717	Parental death; surviving progeny sickly
62	CBG16845	CBG24198	
63	CBG18294	*Cbr-gpc-2*	Weak Let
64	CBG18327	CBG20088	

(*B*) Single RNAi Injections	
Gene	Phenotype(s)
*Cbr-arl-8*	Let; surviving progeny have aberrant oocytes
CBG22317	Aberrant oocytes
*Cbr-nos-2*	Aberrant oocytes, abnormal somatic gonad migration, rarely no germline
CBG04207	Aberrant oocytes
CBG08921	Aberrant oocytes
*Cbr-swd-3.3*	Aberrant oocytes
CBG10091	Proximal oocyte necrosis
CBG13227	Aberrant oocytes; laying oocytes instead of embryos
*Cbr-tpa-1*	Aberrant oocytes
CBG03085	Aberrant proximal oocytes and postpachytene cells
CBG03615	Aberrant proximal oocytes and postpachytene cells
CBG12096	Delayed gametogenesis; delayed self-fertility
*Cbr-air-1.1+1.2*	Parental death; surviving progeny sickly
*Cbr-rpb-3*	
CBG09348	Parental death
CBG09840	
CBG13131	
*Cbr-mop-25.3*	
CBG06213	
*Cbr-mex-3*	
*Cbr-glp-1*	None, but see Rudel and Kimble (2001)
*Cbr-nhr-43*	
CBG02511	
CBG02683	
CBG20835	

Note.—Phenotypes reported are for progeny of injected mothers unless otherwise noted. Egl, egg-laying defective; Let, embryonic lethal; Pvl, protruding vulva; Sma, small adult size; Ste, sterile adult hermaphrodites. (Names of *Cbr-puf* genes based on revision of Liu et al. [2012].) No entry means that no knockdown phenotype was observed.

### *Caenorhabditis briggsae* GLD-1 Targets Are Associated with Germline Developmental Processes

The knockdown phenotypes described above indicated that Cbr-GLD-1 regulates transcripts required for normal germline development. To validate this inference using an alternative approach, we analyzed the Gene Ontology (GO) terms associated with each *C. briggsae* GLD-1-associated transcript (Gene Ontology Consortium 2000). Because *C. briggsae* gene annotations are still incomplete, GO terms were lifted from the *C. elegans* homolog most similar in sequence. We then analyzed the collection for overrepresented terms (Huang et al. 2009). Among the most highly enriched Biological Processes for the Cbr-GLD-1-associated transcripts were reproduction, embryonic development, postembryonic development, anatomical structure development, development ending in birth or hatching, and reproductive process in a multicellular organism (Bonferroni corrected *P* values < 0.02, fold enrichment > 1.25, each term containing >10% input genes; supplementary table S3, Supplementary Material online). The first two of these were also reported as statistically enriched among the GLD-1 targets of *C. elegans* (Jungkamp et al. 2011; Wright et al. 2011). Additionally, enriched Cellular Component and Molecular Function GO terms (not reported in the *C. elegans* studies) include nucleus, intracellular membrane-bounded organelle, nucleic acid binding, and protein binding (supplementary table S3, Supplementary Material online). Three *C. elegans* GLD-1 target-associated GO process terms not enriched among Cbr-GLD-1 targets were cell division, cytokinesis, and cell cycle*.* The differential enrichment of some GO annotations may reflect shifts in the biological roles of GLD-1, but shortcomings in annotations cannot be ignored as an alternative explanation.

### Motif Discovery in *C. briggsae*-GLD-1-Associated Transcripts

*Caenorhabditis elegans* and *C. briggsae* GLD-1 are 85% identical and 91% similar at the amino acid level, and *C. briggsae* GLD-1 can rescue a *C. elegans* GLD-1 null mutation (Beadell et al. 2011). Ce-GLD-1 and Cbr-GLD-1 therefore likely bind very similar ribonucleotide motifs, so strong enrichment of the previously published *C. elegans* GLD-1-binding motifs (GBMs) (the STAR-binding element, or SBE, of Ryder et al. 2004, and the GBM of Wright et al. 2011) is expected within *C. briggsae* GLD-1-associated transcripts. We examined the conservation of binding motifs by examining the putative regulatory untranslated regions (UTRs) of Cbr-GLD-1-associated transcripts. Because the UTRs of *C. briggsae* genes were largely unannotated, we retrieved 100 bp upstream and 250 or 400 bp downstream of coding sequences for *C. briggsae* GLD-1-associated transcripts to approximate an UTR library (Hajarnavis et al. 2004).

**Fig. 3.—:**
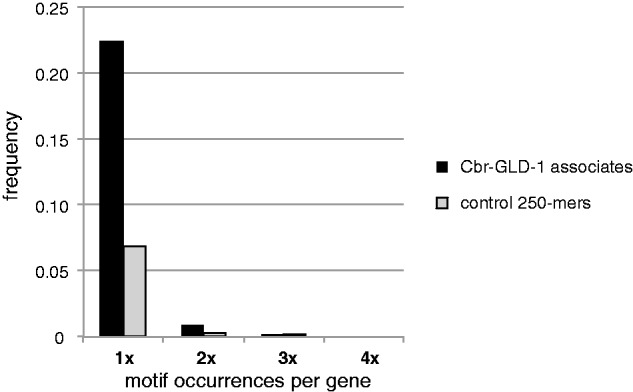
Enrichment of GBMs in Cbr-GLD-1-associated mRNAs. Frequency of two specific hexamers, ACUAAC and ACUCAC, in the 250 bp downstream of the stop codon of the 965 Cbr-GLD-1-associated transcripts or a control set of 10,000 randomly chosen 250-mers from the *Caenorhabditis briggsae* genome assembly cb4 (Ross et al. 2011). These motifs are “strict” instances of the SBE (Ryder et al. 2004) also consistent with the GBM (Wright et al. 2011).

We first examined enrichment for two specific instances of the partially degenerate GLD-1-binding sequence, ACUAAC and ACUCAC, which are “strict” instances of the SBE (Ryder et al. 2004) that are also consistent with the GBM (Wright et al. 2011). In the 250 bp of sequence 3′ of the stop codon of Cbr-GLD-1-associated transcripts, these motifs were found at roughly four times the rate of control fragments of *C. briggsae* genomic DNA (fig. 3). To look more comprehensively, we used the “GBM_finder” script of Wright et al. (2011) to identify motifs associated with *C. elegans* GLD-1 binding (Ryder et al. 2004; Wright et al. 2011) among the upstream and downstream regions of Cbr-GLD-1 targets (supplementary table S4, Supplementary Material online). In total, 76.2% (735/965) of *C. briggsae* targets have flanking sequences that contain motif variants associated with GLD-1 binding in *C. elegans* RIP-chip experiments (i.e., GBM level > 0; Wright et al. 2011; supplementary table S4, Supplementary Material online). Conversely, of a random set of 11,064 complete *C. elegans* transcripts (containing both 5′- and 3′-UTR sequences), 40.1% contained a GLD-1-binding motif with GBM level greater than 0 anywhere in their transcript. These results indicate that most of the Cbr-GLD-1-associated transcripts have the potential to be directly bound. The others may represent opportunistic binding in lysates or indirect binding through protein–protein contacts between Cbr-GLD-1 and other RBPs.

In order to investigate the potential for coregulation of GLD-1-associated transcripts by other RBPs, we also searched de novo for RBP motifs enriched in the 5′- and 3′-flanking sequences of *C. briggsae* GLD-1 targets with three different computational methodologies (D’haeseleer 2006; MacIsaac and Fraenkel 2006; Fan et al. 2009) and then tried to match these motifs to a database of RBP specificities (Cook et al. 2011). Of the 22 nonredundant motifs with the highest information content, seven are consistent with previously published GLD-1-binding sequences (supplementary table S5, Supplementary Material online). Other motifs are similar to RBPs involved in splicing, including Nova2 and SFRS2, which may reflect the similar RNA-binding motif of GLD-1 and the STAR-domain branch-point-binding splicing factor SF1 (Ryder et al. 2004).

### Pan-Metazoan Conservation of a Subset of *C. briggsae* GLD-1-Associated Transcripts

As GLD-1 is found within a nematode-specific expansion of STAR proteins and is unique among characterized STAR proteins for being exclusively cytoplasmic, we asked to what degree the targets of *C. briggsae* GLD-1 are found in nonnematode species. We find that 473 (49%) of the Cbr-GLD-1-associated transcripts have homologs with BLASTP hits from nonnematode taxa in the National Center for Biotechnology Information (NCBI) nonredundant protein database with *e* values less than 1 × 10^−^^10^. In total, 443 targets are conserved in fruit flies (46%), 471 in zebrafish (49%), 456 in chickens (47%), 473 in humans (49%), and 208 in budding yeast (22.5%) (supplementary fig. S5 and table S6, Supplementary Material online). For the 407 Cbr-GLD-1 targets common to bilaterians, significant GO term “functional annotation clusters” were intracellular membrane bound organelle and nucleus; reproductive process; anatomical structure morphogenesis; and multicellular organismal, embryonic, postembryonic, and larval development (Bonferroni-adjusted *P* values < 0.005, fold enrichment > 1.5, >15% input genes for each term). Of the 492 nematode-specific Cbr*-*GLD-1-bound transcripts, we also found that 11 (comprising four small families) have no BLASTP hits to any taxon other than *C. briggsae* with an *e* value <1 × 10^−^^10^ (supplementary table S7, Supplementary Material online). RNAi knockdown of each of these genes either individually or for all genes within each family at once failed to produce observable phenotypes.

### Comparing the Identity of *C. briggsae* and *C. elegans* GLD-1 Targets

**Fig. 4.—:**
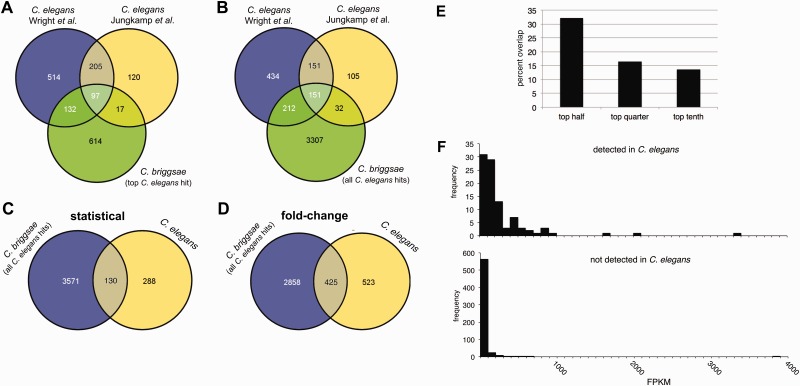
Conservation of GLD-1 targets between *Caenorhabditis briggsae* and *C. elegans*. (*A*) Venn diagram of the overlap of self-reported GLD-1 targets between *C. briggsae* (this work) and *C. elegans* (Jungkamp et al. 2011; Wright et al. 2011), using the top BLASTP hit of each Cbr-GLD-1 target with an *e* value of less than 1 × 10^−10^ against the *C. elegans* proteome. (*B*) Overlap of self-reported GLD-1 targets between *C. briggsae* and *C. elegans*, using all BLASTP hits to each Cbr-GLD-1 target with an *e* value of less than 1 × 10^−10^ against the *C. elegans* proteome. (*C*) Venn diagram of all *C. elegans* homologs of *C. briggsae* GLD-1 targets (as defined statistically in this study) with BLASTP *e* values of less than 1 × 10^−10^ (blue) versus *C. elegans* GLD-1 targets as determined by reanalyzing the Wright et al. (2011) data (yellow). (*D*) Venn diagram of all *C. elegans* homologs of the top 965 Cbr-GLD-1 targets as defined by fold change in anti-GLD-1 versus mock IPs (blue) and the *C. elegans* GLD-1 targets reported in Wright et al. (2011) by fold-change criterion (yellow). (*E*) Degree of interspecies GLD-1 target overlap as a function of enrichment. Self-reported GLD-1 targets for *C. elegans* (Wright et al. 2011) and *C. briggsae* (Beadell et al. 2011) were sorted by fold-change or statistical criteria, respectively, and overlap of different strata was assessed. Overlap existed when a *C. elegans* target was among the set of homologs (with BLASTP score < 10^−10^) of *C. briggsae* targets in the equivalent stratum. For example, 16.5% (39) of the top 25% of *C. elegans* targets were among the 1,239 *C. elegans* genes homologous to the top 25% of *C. briggsae* hits. (*F*) XX young adult expression levels for *C. elegans* orthologs of Cbr-GLD-1-associated transcripts also detected in *C. elegans* by both Wright et al. (2011) and Jungkamp et al. (2011) (top) and those not detected in either *C. elegans* study (bottom).

To compare Ce-GLD-1 and Cbr-GLD-1 target transcripts, we took advantage of two published genome-wide analyses of *C. elegans* GLD-1 targets: Wright et al. (2011), which employed RIP-chip with a functional tagged GLD-1 transgene to call 948 targets, and Jungkamp et al. (2011), which employed iPAR-CLIP on a similar transgenic *C. elegans* strain to identify 439. These two data sets have 68.8% (302/439) of their elements in common. Interspecies comparisons of GLD-1 target sets are complicated by species-limited genes and paralogs. Of the 965 *C. briggsae* GLD-1-associated transcripts identified in this work, 899 have probable homologs in *C. elegans*, using a BLASTP *e* value criterion of less than 1 × 10^−^^10^, 860 of which are unique. This number is consistent with genome-wide homology (Stein et al. 2003). We performed one set of comparisons using the highest-scoring *C. elegans* BLASTP hit of each of these 860 genes to the targets reported by Wright et al. (2011) and Jungkamp et al. (2011) (fig. 4*A*). This resulted in a maximal overlap of approximately one-quarter in both cases, and this low degree of concordance does not increase if we consider only the most highly enriched transcripts from the two studies (fig. 4*E*). When allowing for species-specific paralogs and more distantly related homologs by accepting all *C. elegans* BLASTP hits with *e* values less than 1 × 10^−^^10^, overlap increased to 41–45% (fig. 4*B*), still surprisingly little.

The relatively weak overlap between *C. briggsae* and *C. elegans* GLD-1 targets could be due to the different microarray analysis methods employed in each study. We addressed this possibility in two ways. First, we subjected the preprocessed and normalized microarray data of Wright et al. (2011) to our statistical analysis pipeline (see supplementary material, Supplementary Material online). Comparing the highest scoring *C. elegans* BLASTP hit for each of the possible 860 *C. briggsae* GLD-1-associated mRNAs with the 418 *C. elegans* GLD-1-associated gene products we identified using SAM and EDGE, we find only 14.6% (61/418) overlap. Expanding the comparison to include all *C. elegans* homologs of *C. briggsae* GLD-1 targets, as above, increases the overlap to 31.1% (fig. 4*C*). We also analyzed the *C. briggsae* GLD-1 RIP-chip data with the method of Wright et al. (2011) by examining the anti-GLD-1 IP versus mock IP expression comparison using a fold-change cutoff criterion (supplementary table S9 and fig. S4, Supplementary Material online). 32.4% (282/871) of the highest scoring *C. elegans* BLASTP hits of each of these fold-change gene products overlap with the 948 Ce-GLD-1 targets originally identified in Wright et al. (2011), and the percentage increased to 44.8% when we include all BLASTP hits to *C. briggsae* targets (fig. 4*D*).

Overall, the above comparisons indicate that, even when using very lenient criteria for homology and analysis, over half of Cbr-GLD-1-associated mRNAs were not detected as Ce-GLD-1 targets in two independent studies. We therefore next considered whether *C. elegans* homologs of *C. briggsae* GLD-1 targets may fail to be detected due to low expression levels. If so, then *C. elegans* genes that are not reported as GLD-1 targets, but whose *C. briggsae* homologs were identified as Cbr-GLD-1 targets, will typically be expressed at a lower level than those that were detected in both species. Indeed, when we examine an XX-specific RNAseq data set (Thomas et al. 2012), we see that the vast majority of “nontargets” are expressed at low (but nonzero) levels, whereas those found in both species encompass a wide range of expression levels that are, on average, much higher (fig. 4*F*). By requiring that nontargets exhibit a minimum expression of 100 fragments per kilobase of mapped reads (FPKM), which is true of most conserved targets, the list of 600 candidate *C. briggsae-*positive, *C. elegans-*negative genes (supplementary table S11, Supplementary Material online) collapses to 41. We regard these 41 genes (table 2) as strong candidates for *C. briggsae-*specific GLD-1-associated transcripts. We also note that these putative Cbr-GLD-1-specific targets were not among the 129 *C. elegans* GLD-1-associated transcripts identified in an independent RIP study that appeared after our original analyses (Doh et al. 2013).

**Table 2:** Candidate *Caenorhabditis briggsae-*Specific GLD-1-Associated mRNAs

WormBase Gene No.	Gene Name	Gene Product Notes	Total GBM Score
WBGene0028332	*CBG05982*	Small subunit of serine palmitoyltransferase	0.912
WBGene00033808	*CBG12955*	Domain of unknown function DUF148	1.677
WBGene00032844	*Cbr-his-72*	Histone H3	0.963
WBGene00038583	*Cbr-rbx-1*	RBX ring finger	NA
WBGene00032440	*Cbr-asp-5*	Aspartyl protease	−0.003
WBGene00024012	*Cbr-dod-23*	Downstream of DAF-16	0.743
WBGene00026551	*Cbr-skr-1*	Skp1-related ubiquitin ligase component	0.332
WBGene00031134	*Cbr-lys-2*	Lysozyme	NA
WBGene00035116	*CBG14697*	Proteolipid membrane potential modulator	1.175
WBGene00033455	*Cbr-ran-4*	RAN-associated nuclear transport factor 2	NA
WBGene00036658	*Cbr-vha-3*	Vacuolar H^+^ ATPase, proteolipid subunit	NA
WBGene00027222	*Cbr-pes-9*	Peptidase M20	1.919
WBGene00032414	*CBG11273*		2.331
WBGene00025463	*Cbr-daz-1*	RRM RBP	0.084
WBGene00038288	*CBG18998*	Serine/threonine PP2A reg. subunit B″, subunit β	1.198
WBGene00026678	*Cbr-nlp-40*	Neuropeptide-like	1.178
WBGene00027603	*Cbr-lmp-1*	Lysosome-associated membrane glycoprotein	0.561
WBGene000034566	*Cbr-mdf-2*	HORMA DNA-binding domain, spindle checkpoint	NA
WBGene00026341	*Cbr-fat-4*	Fatty acid desaturase	NA
WBGene00034027	*Cbr-elb-1*	Elongin B, chromosome condensation factor	1.895
WBGene00028676	*Cbr-puf-7*	PUF RBP	NA
WBGene00027257	*Cbr-pas-1*	Proteasome α subunit	2.566
WBGene00025925	*CBG02968*		NA
WBGene00031204	*CBG09646*	Enhancer of rudimentary	1.436
WBGene00025990	*Cbr-taf-13*	TBP-associated transcription factor family	NA
WBGene00024807	*CBG01590*	Centromere protein X	NA
WBGene00026127	*Cbr-tomm-40*	Translocase of outer mitochondrial membrane	NA
WBGene00042785	*Cbr-nos-2*	Nanos RBP	NA
WBGene00031008	*Cbr-cni-1*	Cornichon domain	NA
WBGene00026004	*Cbr-ebp-2*	RP/EB microtubule end-binding protein	NA
WBGene00028195	*Cbr-elo-3*	GNS1/SUR4 fatty acid elongation factor	NA
WBGene00041780	*CBG23419*	Translation elongation factor EF1 α	1.184
WBGene00027913	*Cbr-blos-2*	Biogenesis of lysosome-related organelles complex-1, subunit 2	NA
WBGene00041670	*Cbr-ape-1*	Ankyrin repeats, apoptosis enhancer	0.153
WBGene00025363	*CBG02285*	Leucine-rich repeat	NA
WBGene00026898	*Cbr-dnc-6*	Dynactin complex subunit 6	0.085
WBGene00033866	*Cbr-lir-1*	LIN-26-related	2.800
WBGene00037251	*CBG17687*	Peptidase M20	0.459
WBGene00026895	*CBG04168*	DOMON domain	0.782
WBGene00039610	*Cbr-csc-1*	Chromosome segregation and cytokinesis factor	NA
WBGene00033472	*CBG12532*	Myb DNA-binding domain, associated with splicing	NA

Note.—Only genes whose *C. elegans* homolog is expressed abundantly are included (see main text for discussion). GBM scores are totals from GBM_finder motifs in the 100 bp upstream or 250 bp downstream of the annotated start and stop codons of each gene model in WormBase. Positive values predict association with GLD-1, negative values nonassociation. NA, no motifs found.

Among the 41 *C. briggsae* GLD-1 targets whose *C. elegans* homologs are well expressed, yet not associated with *C. elegans* GLD-1, are a number of characterized germline transcripts (supplementary table S11, Supplementary Material online). These include those encoding the RBPs DAZ-1, NOS-2, and PUF-7, the embryonic histone HIS-72, and the meiotic regulator MDF-2. Noteworthy transcripts associated with GLD-1 in *C. elegans* but not detected in *C. briggsae* include *gld-1* itself, the p53 homolog *cep-1*, the E-type cyclin *cye-1*, the Bicaudal C homolog *gld-3*, the P-granule protein *pgl-1*, and the nanos homolog *nos-1* (supplementary table S11, Supplementary Material online).

### Features of Putative Cbr-GLD-1-Specific Target Transcripts

Of the 41 *C. briggsae*-specific GLD-associated transcripts described above, 18 have total GBM scores (Wright et al. 2011) for the 100 bp upstream and 250 bp downstream flanking sequences greater than 1, whereas one has a weakly negative score (table 2 and supplementary table S12, Supplementary Material online). This suggests that roughly half of the 41 candidates may be directly bound by Cbr-GLD-1.

**Fig. 5.—:**
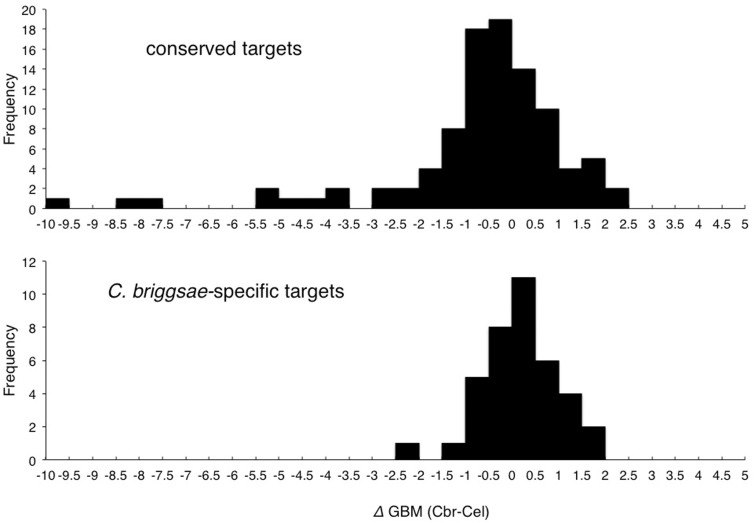
Relationship between GBMs and species-specific GLD-1 association. Each histogram depicts the difference in total GBM density (deltaGBM) between *Caenorhabditis elegans* and *C. briggsae* ortholog pairs. Only genes with annotated *C. elegans* 5′- and 3′-UTRs are included. Upper panel: Cases where both homologs were identified as GLD-1 targets. Lower panel: Cases where the transcript is associated with Cbr-GLD-1 in this study, but whose *C. elegans* ortholog is not reported as associated with Cel-GLD-1 and is also expressed at over 100 FPKM in XX *C. elegans* animals. Omitting the long left tail of the distribution for the conserved targets, the remaining Cbr-GLD-1-specific transcripts still have a significantly higher (deltaGBM values; two-tailed *T*-test, *P = *0.035).

We next examined whether the presence of GBMs (Wright et al. 2011) could explain the apparent Cbr-GLD-1-specific association of these transcripts. We used GBM_finder (Wright et al. 2011) to find candidate GLD-1-binding sites in these open reading frame-flanking sequences (i.e., approximations of UTRs) of both conserved and Cbr-GLD-1-specific associated transcripts. We then calculated the difference in overall GBM score for each ortholog pair (deltaGBM), with the expectation that there would be little overall difference in the 97 conserved targets (i.e., deltaGBM should be near zero on average), but a higher GBM score in *C. briggsae* for the 41 Cbr-GLD-1-specific associates. As shown in figure 5, there is a significantly greater deltaGBM value for homologs with Cbr-GLD-1-specific association than for those with conserved association, and it is in the direction expected (i.e., the *C. briggsae* scores are higher on average). Though the effect is modest, it is consistent with changes in the density of known GBMs contributing to species-specificity of GLD-1 association. In addition, the conserved targets include some cases where the *C. briggsae* homolog’s GBM score is much lower than that of its *C. elegans* counterpart, despite strong evidence of being Cbr-GLD-1-associated (fig. 5, upper panel, values < 3.0). This may reflect the fact that the GBM_finder software was trained using *C. elegans* transcripts. Alternatively, it may indicate that there are species-specific motifs that are important for GLD-1-binding, or indirect binding through other protein cofactors that obviate the need for a GBM entirely.

### Features of Conserved GLD-1 Target Transcripts

The 97 GLD-1 targets in common between *C. briggsae* and *C. elegans* include (in *C. elegans* nomenclature) the H1-like histones *hil-4* and *hil-5*; the nuclear importins *ima-1*, *ima-2*, and *ima-3*; the DNA replication licensing factors *mcm-3*, *mcm-5*, and *mcm-7*; the cyclin B family members *cyb-*2 and *cyb-3*; and the Argonautes *ergo-1*, *prg-1*, *ppw-*2, and *wago-4*. Previously confirmed or suggested *C. elegans* GLD-1 targets *cpg-1* and *-2*, *gna-2*, *glp-1*, *mes-3*, *mex-3*, *oma-1/*2, *pal-1*, *pie-1*, *rme-2*, and *spn-4* are also bound by *C. briggsae* GLD-1.

To learn more about the attributes of the conserved targets, we analyzed the GBM content of the 85 orthologous pairs of GLD-1-associated transcripts for which UTR or UTR-like sequences were available (supplementary table S13, Supplementary Material online). We sought to determine whether GBMs tend to remain in the same place and number when overall binding is conserved. We probed the longest transcripts in the case of *C. elegans*, or proxy transcripts with the same length UTRs in the case of *C. briggsae* putative orthologs. We classified an orthologous UTR as containing evolutionarily conserved GBMs if 50% or more of the GBMs in any particular UTR fell within 15 bp of a GBM in the orthologous UTR, settling on a 15-bp cutoff after initial rounds of data examination.

**Fig. 6.—:**
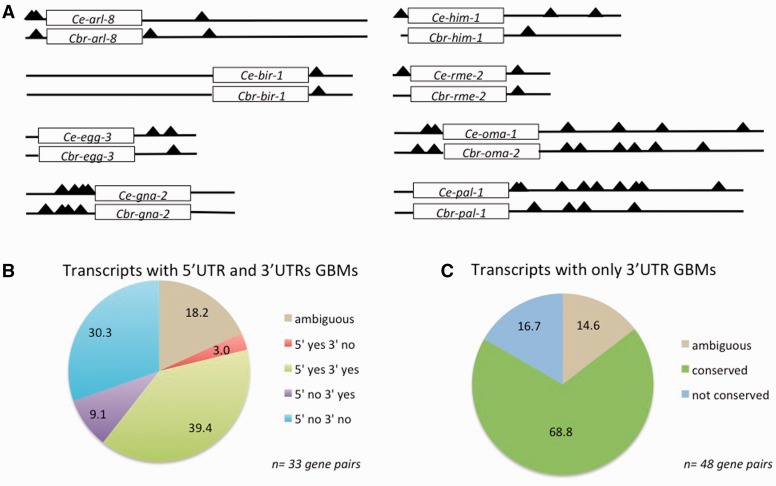
Conservation of GLD-1-binding sites among orthologous GLD-1 targets. (*A*) Location of GBMs (black triangles, as given in supplementary table S13, Supplementary Material online) within the untranlslated regions of eight representative orthologous GLD-1 target transcript pairs, illustrating different patterns of GBM conservation. For each transcript, the rectangle represents the open reading frame (not drawn to scale), whereas the lines to the left and right represent the 5′- and 3′-UTRs, respectively, and are drawn to scale. (*B*, *C*) Bulk analysis of GBM conservation in transcripts that are GLD-1 associated in both *Caenorhabditis elegans* and *C. briggsae.* (*B*) Inferred conservation of GBMs in orthologous genes for which at least one ortholog of each pair possesses a GBM in its 5′-UTR. (*C*) Inferred conservation status of GBMs for cases where both orthologs only possess GBMs in their 3′-UTR. In both (*B*) and (*C*), “ambiguous” cases include orthologs containing GBMs beyond our 15-bp cutoff (though these sites may be identical or nearly identical in base pair composition) and/or where less than 50% of the sites in any particular UTR have a counterpart in the orthologous UTR. “yes” and “no” indicate conservation or nonconservation, respectively, of GBMs in the indicated region of the transcript. Four ortholog pairs in which one member completely lacks identifiable GBMs are not included in this figure (see text).

We find that GBM number and location are often conserved (fig. 6*A*). We defined a set of conserved GLD-1-associated transcripts with both 5′- and 3′-UTR GBMs in at least one ortholog (33, or 39% of the total gene pairs; fig. 6*B*) and another where neither has a 5′-UTR GBM of Wright et al. “level” score greater than 0 (a measure of significant GLD-1 association in that study), but where both have 3′-UTR GBMs (48, or 56% of total gene pairs; fig. 6*C*). In both cases, the majority of orthologs have conservation of GBM presence and location given our criteria: Overall, we find that 47/85 (55.3%) gene pairs demonstrate GBM site conservation. In total, 25 pairs (29.4%) do not, whereas another 13 pairs (15.3%) have GBMs somewhat indicative of evolutionary conservation but also do not meet our criteria (ambiguous), containing GBMs just beyond the 15-bp cutoff and/or less than 50% of the sites in any particular UTR with a counterpart in the orthologous UTR (supplementary material and table S13, Supplementary Material online). Orthologs of transcripts in the 5′ + 3′ GBM category are about twice as likely to lack conserved GBMs at either end of the transcript as those with only 3′-UTR GBMs (fig. 6). This suggests that the presence of GBMs in the 5′-UTR of a transcript is associated with more dynamic evolution, but the trend is not significant (at the *P = *0.05 level by a chi-square test).

A final 4 of the 85 pairs of orthologous, conserved GLD-1 targets contain no GBM in either one or both members of the gene pair in either UTR. For example, the Y43H11AL.1/CBG07029 pair does not possess any GBMs in any of their 5′- or 3′-UTRs. These transcripts may be conserved indirect binders of GLD-1, perhaps through association with ribonucleoprotein bodies in the nematode germline, with which GLD-1 is known to associate in part (Schisa 2012). In the C02B10.2\CBG19979 gene pair, the *C. elegans* gene (C02B10.2) possesses five 3′-UTR GBMs, but its *C. briggsae* counterpart CBG19979 does not have any GBMs in either UTRs. Similarly, *C. elegans* Y110A2AR.1 and *dna-2* each have two GBM motifs within their 3′-UTRs, but their putative *C. briggsae* orthologs have no GBMs in either proxy UTR (supplementary table S13, Supplementary Material online). These may be examples of indirect GLD-1 binders that happen to contain nonfunctional GLD-1 motifs within the *C. elegans* ortholog, or perhaps instead showcase differential GLD-1-binding site loss or gain.

### Many Cbr-GLD-1 Targets Encoded by X-Linked Genes

Interestingly, we also find that more *C. briggsae* GLD-1 targets are encoded by X-linked genes than expected by chance (1.4-fold enriched, Bonferroni-corrected χ^2^ *P = *1.4 × 10^−^^6^). This enrichment is not true of *C. elegans* GLD-1 targets, which, as for germline transcripts in general, are actually rarely X-linked (e.g., Fong et al. 2002; Reinke et al. 2004). Only 4/435 Jungkamp et al. (2011) and 32/948 Wright et al. (2011) targets are found on the X chromosome in *C. elegans*, whereas 200/955 GLD-1-associated genes with chromosome assignments are X-linked in *C*. *briggsae*.

## Discussion

Members of the STAR-domain family of RBPs are found throughout eukaryotes. They regulate the fate of RNAs in the nucleus and cytoplasm and perform a range of biological functions including translational repression, alternative splicing, and RNA nuclear export for processes such as cell division, apoptosis, cell differentiation, and gametogenesis (e.g., Lo and Frasch 1997; Zaffran et al. 1997; Pilotte et al. 2001; Nabel-Rosen et al. 2002; Wu et al. 2002; Di Fruscio et al. 2003; Ohno et al. 2008; Iijima et al. 2011; Rodrigues et al. 2012; Wang et al. 2013). We have used an in vivo genome-wide approach to identify mRNA targets of the STAR protein GLD-1 in adult hermaphrodite *C. briggsae* nematodes. This allows the first comparison of RBP–protein complexes in closely related animals.

### The GLD-1/Quaking/How Clade of STAR-Domain Proteins Was Present in the Metazoan Ancestor

The large size of the STAR domain (∼200 amino acids) allowed us to resolve the relationships of GLD-1-related proteins across opisthokonts (fig. 1). GLD-1 lies in a large, nematode-specific clade related to deuterostome Quaking and arthropod How/Who. A second STAR-domain clade contains SAM68/SLM homologs and also maintains clear phyla-specific subfamilies. The closer phylogenetic relationship between GLD-1 and Quaking versus SAM68 is consistent with the similarity in binding motifs between the former (Ryder and Williamson 2004; Galarneau and Richard 2009). However, whereas Quaking and How are nuclear splicing regulators, GLD-1 is cytoplasmic and engages in translational repression. The high proportion of *C. briggsae* GLD-1 targets that seem to be nematode-specific may reflect unique roles of GLD-1 as a cytoplasmic translational repressor for controlling nematode development. As expected, all taxa in our analysis possess homologs of the splicing factor SF1, and nonanimal outgroups possess only this single STAR-domain protein. The branch point-like binding motif of many STAR-domain proteins likely reflects the role of its ancestor in splicing (Ryder et al. 2004). The nonbilaterian metazoans, Hydra and Nematostella (phylum Cnidaria) and Trichoplax (phylum Placazoa), possess an SF1 homolog and proteins that fall as outgroups to both the SAM68-like and Quaking/How-Who/GLD-1 clades. Given these relationships, we posit that the opisthokont ancestor possessed a single STAR protein, likely a splicing factor, and that metazoans experienced an expansion of STAR-domain proteins from 1 to 3.

### Cbr-GLD-1 Associates with Hundreds of mRNAs

We have identified 965 mRNAs consistently associated with *C. briggsae* GLD-1. The RIP-chip method cannot distinguish between direct versus indirect linkage to GLD-1, and the germ line is rich in mRNAs and RBPs with which GLD-1 might associate (Lee and Schedl 2006; Schisa 2012). Nevertheless, these transcripts are enriched for known GBMs and display partial overlap with iPAR-CLIP results from *C. elegans* (Jungkamp et al. 2011), which include only direct targets. This suggests that GLD-1 directly regulates hundreds of mRNAs in *C. briggsae*, as in *C. elegans*. GLD-1 thus appears to be a “broad-spectrum” RBP. Similarly, in the yeast *Saccharomyces cerevisiae*, Hogan et al. (2008) found that 9 of 46 RBPs examined regulate 10% or more of transcripts in that genome. Current work continues to characterize the prevalence and potency of posttranscriptional regulation by RBPs (Klass et al. 2013; Ray et al. 2013). In the nematode germline and early embryo, GLD-1 may help to orchestrate layers of RBP-mediated gene regulation, as both “developmental” and “housekeeping” RBP transcripts are conserved GLD-1 targets between *C. elegans* and *C. briggsae*.

### Cbr-GLD-1 Regulates mRNAs Required for Multiple Aspects of Germline Development

Among the GLD-1 targets common to both *C. briggsae* and *C. elegans*, GO terms that are significantly enriched are cell division, cell fate commitment, DNA metabolic process, oogenesis, and embryonic pattern specification. This is consistent with a common role for GLD-1 in regulating gene expression in germline mitotic cells, early-stage oocytes, and the early nematode embryo (Jones et al. 1996; Nayak et al. 2005). As a more direct validation of germ line roles for Cbr-GLD-1-associated mRNAs, we used RNAi to knock down expression of over a hundred of them. The frequency of germline phenotypes was roughly 25%, comparable to that observed in a similar injection-based screen of known germline genes in *C. elegans* (Colaiacovo et al. 2002), and much greater than observed in whole-genome knockdown studies (e.g., Simmer et al. 2003). Further, almost all experiments that produced an observable phenotype affected the germ line. The most common phenotype was defective oogenesis, including unusual shapes and/or cytoplasmic constitutions, malformation of the most proximal oocyte consistent with its unique meiotic maturation status (McCarter et al. 1999), and slow ovulation/fertilization despite the presence of normal looking oocytes and sperm. These phenotypes are consistent with a conserved role for GLD-1 in regulating oocyte differentiation.

### Motifs within the Flanking Sequences of Putative Cbr-GLD-1 Targets

Published GBMs are enriched in Cbr-GLD-1-associated transcripts using both GLD-1-specific and de novo motif finding algorithms. However, GLD-1 binds to its targets as a homodimer, and a single SBE/GBM, though sufficient for high affinity interaction, can only accommodate the binding of one GLD-1 molecule (Ryder et al. 2004; Beuck et al. 2010; Teplova et al. 2013). The second protomer of the GLD-1 homodimer might bind a “half-site,” an AT-rich, degenerate SBE/GBM-like motif that can be found at variable distances away from the full SBE/GBM (Ryder et al. 2004; Galarneau and Richard 2009; Carmel et al. 2010). The strength of GLD-1 binding is also thought to be proportional to the number of GLD-1 homodimers that can associate through multiple binding sites within a given transcript (Wright et al. 2011). Correctly predicting the overall strength of target association with GLD-1, then, likely needs to incorporate the SBE number, potential half sites, as well as their three-dimensional accessibility to GLD-1 proteins (supplementary material, Supplementary Material online; Lehmann-Blount and Williamson 2005; Brümmer et al. 2013). These complexities create limitations for the analyses presented here, and suggest that future comparative work would benefit from the PAR-CLIP method, which selectively identifies directly interacting mRNAs and defines their RBP-binding sites (Hafner et al. 2010).

### GLD-1 Has Both Conserved and Dynamic Targets

GLD-1 orthologs exist in all *Caenorhabditis* nematode species examined to date (Beadell et al. 2011) and have highly similar sequences, very similar protein expression patterns, and share some loss-of-function phenotypes (Nayak et al. 2005; Beadell et al. 2011). Further, a construct containing *C. briggsae gld-1* regulatory and coding sequences fully rescues a *C. elegans* GLD-1 null mutation (Beadell et al. 2011). Despite these similarities, we detect only modest overlap between *C. elegans* and *C. briggsae* GLD-1-associated transcripts. Low cross-species target overlap does not seem to be simply an artifact of comparing different techniques or employing different analysis methods, although this may contribute to discordance. Instead, transcript abundance data suggest that a large number of the Cbr-GLD-1+/Ce-GLD− homologs are false negatives in the *C. elegans* studies. If true, then there may be many more targets yet to be discovered, even in *C. elegans*.

Loss-of-function mutants of *C. elegans* and *C. briggsae gld-1* share a tumorous germline phenotype (Francis, Barton, et al. 1995; Beadell et al. 2011), consistent with a conserved role in committing developing germ cells to meiosis. However, as noted above, loss of *gld-1* function has opposite effects on germline sex determination in these two hermaphroditic species, and no effect on sex in gonochoristic relatives (Francis, Maine, et al. 1995; Nayak et al. 2005; Beadell et al. 2011). GLD-1-associated mRNAs conserved between the two species represent good candidates for the meiotic role shared between species, and they include genes known to regulate key steps of the cell cycle (see supplementary table S10, Supplementary Material online).

We also note an excess of X-linked transcripts in our Cbr-GLD-1 associates. Given the silencing of X-linked gene expression in the *C. elegans* hermaphrodite germ line (Pirrotta 2002), even proportional representation of X-linked targets would be surprising. Although it is possible this is a technical artifact (e.g., due to somatic transcripts binding postlysis to GLD-1), this should also have occurred in the *C. elegans* studies, but did not. The physical map of the *C. briggsae* X chromosome has been carefully assembled (Hillier et al. 2007; Ross et al. 2011), so the result cannot be explained by chromosomal misassignments. Taken at face value, the presence of many X-linked transcripts associated with *C briggsae* GLD-1 may point to an interesting biological difference in the way that *C. briggsae* and *C. elegans* regulate transcription from their sex chromosome in the germline and/or the early embryo.

### RBPs and Evolution

Though the contributions that changes in transcription factor binding sites across species make to evolutionary divergence have received abundant attention in the literature, substantially less has been paid to the potential for changes in the *cis*-regulatory sequences of RNA molecules to shape evolution. It only seems logical, though, that changes in the regulation of mRNA molecules can lead to the expression of variation upon which selection acts. Previous studies have revealed that homologous PUF family RBPs, for instance, in distantly related eukaryotes bind distinct target mRNAs (e.g., Gerber et al. 2004, 2006). This implies that RBP targets can diverge over long periods of time, but the phylogenetic coarseness of these studies obscures the process of change. Our comparison of GLD-1-associated mRNAs in *C. briggsae* and *C. elegans* involves much closer relatives, yet we still see evidence of divergence. It should be noted that *C. elegans* and *C. briggsae* are both unusual among *Caenorhabditis* nematodes in possessing an androdioecious (hermaphrodites/males) mating system, which they independently evolved from different male/female ancestors (Kiontke et al. 2004). This may make attributes of their XX germ cells atypically diverged due to incongruent mechanisms of adaptation (True and Haag 2001). However, GLD-1 has only been implicated in the regulation of a handful of sex determination gene products in each species (Jan et al. 1999; Beadell et al. 2011), so the shifts to hermaphroditism seem unlikely to explain our results. *Caenorhabditis* nematodes do experience a notoriously fast rate of nucleotide substitution (Cutter et al. 2009), and this may cause a baseline level of target turnover that is of little functional consequence. Alternatively, the fine-scale regulation of GLD-1 and/or its targets may differ between *C. briggsae* and *C. elegans* in ways that are important yet cryptic. The C-terminus seems to be the predominant site of posttranslational regulation for STAR proteins (Sette 2010) and also contains the most divergent amino acid sites among GLD-1 proteins of different *Caenorhabditis* species (Beadell et al. 2011).

The function and evolution of GLD-1-binding sites among orthologous/homologous transcripts in *C. briggsae* and *C. elegans* have been characterized for one gene, *tra-2* (Goodwin et al. 1993; Beadell et al. 2011). Here, differential association appears to be related to a recent expansion of GLD-1-binding elements in the *C. elegans tra-2* 3′-UTR, mediated by a tandem duplication in that 3′-UTR. The *tra-2* mRNA also provides an example of a transcript that is regulated by different RBPs in different tissues: GLD-1 represses the translation of *C. elegans tra-2* in the germline, but SUP-26, which is unrelated to GLD-1, carries out this function in the soma using the same 3′-UTR tandem duplication (Mapes et al. 2010). This is reminiscent of the metazoan posterior fate determinant caudal/*pal-1*, whose transcript is regulated in the *Caenorhabditis* germline/early embryo by GLD-1 (and by PUF-8), but by the DNA/RNA-binding protein Bicoid (and Pumilio, a PUF-8 homolog) in *Drosophila* (Dubnau and Struhl 1996; Rivera-Pomar et al. 1996; Mootz et al. 2004; Gerber et al. 2006; Mainpal et al. 2011).

Among the likely species-specific Cbr-GLD-1 targets identified in this study, *daz-1* is especially intriguing. It encodes a broadly conserved, germ line-specific RBP associated with sperm development in most phyla (Haag 2001), but in *C. elegans* it is required for oogenesis instead (Karashima et al. 2000, Maruyama et al. 2005). Further, Otori et al. (2006) found that in *C. briggsae*, *daz-1* loss-of-function animals make only sperm in a female somatic gonad. This phenotype is strikingly similar to that of *Cbr-gld-1* (Nayak et al. 2005; Beadell et al. 2011), and we now have evidence for species-specific association of the *daz-1* mRNA with Cbr-GLD-1. That the phenotypes are the same is inconsistent with Cbr-GLD-1 acting as a repressor of *Cbr-daz-1* activity. However, some transcripts are stabilized by GLD-1 (Lee and Schedl 2004; Scheckel et al. 2012), and this may be relevant here.

We also note that the *cep-1* p53 tumor suppressor homolog was identified as a Cel-GLD-1-specific target, but was not associated with Cbr-GLD-1. This is intriguing, as *cep-1* is known to be required for programmed cell death in cells of the XX *C. elegans* germ line that experience DNA damage or meiotic failure (Schumacher et al. 2005). Cel-GLD-1 limits *cep-1* activity, preventing excessive cell death. As much of this pathway is likely to be conserved in *C. briggsae*, this is surprising.

Probing the dynamics of RBPs and their targets during evolution is likely to become more prevalent as methods for isolating RNA and their binding proteins become easier and as more RNA regulatory molecules (whether they be proteins or RNAs themselves) are characterized. Evolution at the posttranscriptional level of gene regulation may be particularly important where gene regulation through transcription is disfavored, such as in meiotic germ cells, early embryos, and neuronal synapses. Future work in the Caenorhabtis GLD-1 system should therefore focus on the evolutionary dynamics of specific GBMs and their relevance to development and phenotypic divergence.

## Materials and Methods

### Phylogenetics

We used BLASTP 2.2.20 (Altschul et al. 1997) and the full length Cbr-GLD-1 query to search the NCBI nonredundant protein RefSeq database using default parameters. Hits from representative opisthokonts with *e* values less than 1 × 10^−^^10^ were retained, and partial sequences (lacking the initial methionine or sequences <100 amino acids) and nearly identical sequences were removed. The resulting 96 sequences (supplementary table S1, Supplementary Material online) were aligned using ClustalX 2.0.11 (Larkin et al. 2007). We trimmed sequences N-terminal and C-terminal to the conserved KH and Qua1 and Qua2 domains iteratively to find the maximal alignable protein fragment. We used the ClustalX option “Iterate each alignment step,” which significantly improved the alignment, and we removed all gaps before each realignment. The Pairwise Alignment gap-opening penalty was changed to 35 (from 10) and the gap extension penalty to 0.75 (from 0.1). The final total alignment length was 451 amino acids long.

A Bayesian phylogeny was inferred using MrBayes (Ronquist and Huelsenbeck 2003) with the following parameters: 1 million generations, burn-in period of 25% of generations, rate parameter set to “adgamma” (in which rates vary across sites according to the gamma distribution but the rate at each site depends in part on the rates at adjacent sites), and the amino acid model “mixed.” All other run parameters were default settings. From the output, we determined that cold chains were reasonably mobile, the standard deviation of split frequencies descended to 0.01, and that the potential scale reduction factor was near 1 for all parameters and for partition branch lengths. We examined the output plot of generation versus log likelihood to find that stationarity was reached. Phylogenetic trees were edited using Dendroscope (Huson et al. 2007).

### Microarray Design and Analysis of Data

We modified the oligonucleotide probe set for all predicted *C. briggsae* protein-coding genes (WormBase release cb25.agp8) of Yanai and Hunter (2009), in which probes are 3′-biased and 50–60 bases long. The starting probe set covered 100% of genes with at least one probe, 98% with at least two probes, and 2.6% with three probes. We added third probes where absent for the following categories of genes: Those potentially involved in sex determination (Ellis and Schedl 2007); *Cbr-puf* genes (Liu et al. 2012); some genes encoding homologs of *C. elegans* RBPs (Lee and Schedl 2006); and some genes involved in RNAi/microRNA processing. Five “positive control” genes (orthologs of known *C. elegans* GLD-1 targets: *Cbr-oma-1/2*, *Cbr-rme-2*, *Cbr-glp-1*, *Cbr-mes-3*, and *Cbr-pal-1*) and ten “negative control” genes (randomly chosen genes with somatic-specific or somatic-enriched expression as determined from WormBase) were chosen a priori, and third probes were added to the arrays where needed for these controls. Microarrays were synthesized using the Agilent eArray system.

RIP-chip was performed on extracts of *C. briggsae* hermaphrodites synchronized at the young adult stage using a polyclonal chicken anti-GLD-1 antibody as described, (supplementary fig. S1, Supplementary Material online; Beadell et al. 2011). To assay the specificity of anti-GLD-1 IPs here, we used gel mobility shift assays and quantitative real-time PCR to verify the binding and enrichment of positive control Cbr-GLD-1 targets *Cbr-rme-2* and *Cbr-oma* (the single *C. briggsae* ortholog of *C. elegans* paralogs *Ce-oma-1* and *Ce-oma-2*) and nonenrichment of negative control transcripts *Cbr-nol-1* and *Cbr-actin*, the latter obtained with a pan-actin primer set (Nayak et al. 2005; Beadell et al. 2011; fig. 2*C*, and data not shown). Immunoblots with a second anti-GLD-1 antibody also confirmed the specific IP of Cbr-GLD-1 (Beadell et al. 2011, data not shown). Our microarray analysis also revealed enrichment of the five above “positive control” genes and no enrichment of negative control genes.

RNA recovered from the IPs was amplified and labeled with the Kreatech aRNA labeling kit by the Microarray Core Facility at Washington University, St Louis for microarray analysis. cDNA was hybridized to eight Agilent 44K dual-color arrays: Five arrays for anti-GLD-1 versus anti-IgY mock IP expression comparisons and three arrays for anti-GLD-1 IP versus total input mRNA expression comparisons, together from five total biological replicates. Both expression comparisons incorporated dye swaps. Quality controls included examining diagnostic plots of foreground and background intensities versus spatial array coordinates and MA plots of transformed but unnormalized data for each array (supplementary fig. S2, Supplementary Material online, and data not shown). We also created normal probability plots of raw and log_2_-transformed data from each experiment to assess normality and trends (supplementary fig. S2, Supplementary Material online, and data not shown). We did not filter out or differentially weigh any values. Details regarding background subtraction and normalization are given in the main text and in supplementary figure S3, Supplementary Material online. We chose to normalize with eCADS as it integrates intensity measurements within dye-swaps across arrays without forcing particular distributions of measurements (Dabney and Storey 2007). Differential gene expression was inferred using SAM (Tusher et al. 2001) and EDGE (Leek et al. 2006). In SAM, we used 200 permutations; in EDGE, we selected 200 iterations. Venn diagrams were created with VENNY (Oliveros 2007).

### Gel Mobility Shift Assay

*Caenorhabditis elegans* GLD-1 can bind to the 5′-UTR, 5′-coding region, and 3′-UTR of *C. elegans rme-2* (Lee and Schedl 2001; Jungkamp et al. 2011; Wright et al. 2011). We identified multiple potential GBMs in exon 1 of *C. briggsae rme-2*. A maltose-binding protein (MBP) fusion protein to the STAR domain of Cbr-GLD-1 (amino acids 135–329) was produced from a derivative of a plasmid encoding the analogous *C. elegans* construct as previously described (Ryder et al. 2004; courtesy of S. Kwan and J. Williamson). Exon 1 of *Cbr-rme-2* was amplified from *C. briggsae* cDNA, and gel-purified template DNA was transcribed in vitro using the T7 Megascript kit (Ambion). Transcripts were end-labeled with [γ-32P] ATP and polynucleotide kinase. Twenty femtomoles of labeled RNA was incubated for 2 h with varying concentrations of MBP-Cbr-GLD-1 STAR and resolved on native polyacrylamide gels as described (Chritton and Wickens 2010).

### RNAi Knockdown of Putative Cbr-GLD-1 Targets

We used *C. briggsae* gene predictions from WormBase (build CB3) and the NCBI primer-designing tool (http://www.ncbi.nlm.nih.gov/tools/primer-blast, last accessed December 31, 2014) to design primers that yield unique PCR products 400–900 bp in length. T7 RNA polymerase promoter-tailed versions of each primer were used to amplify PCR products from either *C. briggsae* genomic DNA or cDNA. Single, appropriately sized products were used to produce double-stranded RNA (dsRNA) with the MegaScript T7 RNA kit (Ambion). dsRNAs, 1–3 mg/ml, were injected into the gut of adult *C. briggsae* hermaphrodites raised at 20 °C. A first round of injections was conducted using pairs of gene products. Paralogs or members of gene families were coinjected when identified. Gene products known to genetically interact with one another were injected separately. Gene products expected a priori to cause lethality or sterility were reinjected as singletons, as were gene products originally injected in pairs that resulted in these phenotypes unexpectedly. The remaining gene pairs were determined randomly. Injected animals were recovered to 20 °C, moved to a fresh plate in approximately 12 h, and their remaining progenies were scored 3–5 days later (i.e., as young adults). Gross abnormalities (motility, body shape, etc.) were assayed using the stereo dissecting microscope, which germline abnormalities were inferred from differential interference contrast (DIC) microscopy of roughly 20 animals per injection. Phenotypes were typically observed in one-third of progeny.

### GO Analysis

We used *C. elegans* homologs of *C. briggsae* genes to abstract information about *C. briggsae* GLD-1 target genes when available. We obtained a database of each species’ proteins from WormBase (brigpep.WS222.fa and wormpep.WS222.fa) to identify the single best BLASTP hit to each of the 965 Cbr-GLD-1 targets identified here against the *C. elegans* proteome with an *e* value score lower than e-10. We used the DAVID Bioinformatics Resources 6.7 (Huang et al. 2009) to assign and analyze associated GO terms (Gene Ontology Consortium 2000) using the following parameters: For Clustering, “Kappa Similarity,” Similarity Term Overlap = 5, and Similarity Threshold = 0.7; and “Classification,” Initial Group Membership = 10, Final Group Membership = 10, and Multiple Linkage Threshold = 0.7.

### Motif Discovery

Few *C. briggsae* gene models contain experimentally determined UTRs. In order to identify nucleic acid sequences common to the regulatory sequences of *C. briggsae* GLD-1 targets, we used the fact that in *C. elegans*, approximately 80% of 3′-UTRs were found to be no longer than 400 bp and approximately 67% no longer than 250 bp (Hajarnavis et al. 2004). As most *C. elegans* 5′-UTRs are short (Rhoads et al. 2006), we obtained 100 bp upstream and either 250 or 400 bp downstream of each of the 965 *C. briggsae* GLD-1 targets (when possible) using WormMart (release WS222). Motif-finding analyses were run on both repeat-masked and nonrepeat-masked sequences (Smit et al. 1996).

To search the *C. briggsae* UTR-like sequences for overrepresented motifs, we used Weeder (Pavesi et al. 2004; Pavesi and Pesole 2006); Sombrero (Mahony et al. 2005), with a Markov background model using 250 repeat-masked bp downstream of the stop codon of 11,000 randomly chosen *C. briggsae* genes; nMICA (Down and Hubbard 2005), after evaluating different backgrounds using the same input file as for Sombrero; and MEME (Bailey et al. 2006), with various expected motif frequencies, widths, and the backgrounds (default, Markov models based on the *C. elegans* genome, or Markov model backgrounds based on the background file used in Sombrero and nMICA). These methods were able to detect the SL1 splicing signal on the computationally delimited “5′-UTR-like” sequences flanking *C. briggsae* GLD-1 targets and also the polyadenylation signal on the “3′-UTR-like” sequences. Redundant motifs and those with low information content (e.g., sequences with 7/8 degenerate positions or with homopolymeric sequences) were eliminated by visual inspection. Matches to the remaining motifs were found in the RBP database (RBPDB) of Cook et al. (2011) using the “Similarity Matching” feature of “STAMP” (Mahony and Benos 2007).

### Motif Comparisons between Orthologs

Position and number of motifs consistent with the empirically derived GBM (Ryder et al. 2004; Wright et al. 2011) were determined applying Wright et al.’s GBM_finder script to all 96 putatively orthologous pairs of genes we identify as robustly associated with GLD-1 in both *C. elegans* and *C. briggsae* (supplementary table S13, Supplementary Material online). The UTRs of the longest annotated transcripts were used for *C. elegans*, whereas proxies for UTRs were created for *C. briggsae* orthologs. The latter process involved two steps. Initially, we acquired 100 and 250 bp of sequence upstream and downstream of each start and stop codon, respectively. Subsequently, these proxies were either elongated or trimmed as required to match the UTR length of each *C. elegans* counterpart. Eight *C. elegans* genes had unannotated UTRs, and were dropped from the data set, leaving 84 orthologous gene pairs for which both 5′-UTR/upstream sequence and 3′-UTR/downstream sequence were available for analysis.

We classified an orthologous UTR among gene pairs as containing evolutionarily conserved GBMs if 50% or more of the GBMs in any particular UTR fell within 15 bp of a GBM in the orthologous UTR, settling on a 15-bp cutoff after an initial round of data examination. As the GBM is only 7 nt long and degenerate at most positions (Ryder and Williamson 2004; Wright et al. 2011), is A-T-rich in a genome whose noncoding sequences are also very A-T rich, and that nematode sequence evolution is notoriously fast (Cutter et al. 2009), we did not consider base pair identity as a characteristic by which to define conservation (though many examples of GBM conservation do possess identical or nearly identical sequences; supplementary table S13, Supplementary Material online).

## Supplementary Material

Supplementary material, figures S1–S4, and tables S1–S13 are available at *Genome Biology and Evolution* online (http://www.gbe.oxfordjournals.org/).

Supplementary Data
