# Supplementary material for: Evolutionary Dynamics of GLD-1–mRNA Complexes in Caenorhabditis Nematodes
Source: Genome Biol Evol. 2014 Dec 9;7(1):314–35. doi: 10.1093/gbe/evu272 (PMC4316625; doi:10.1093/gbe/evu272)
Supplement: Supplementary Data [file supp_7_1_314__index.html]

Evolutionary dynamics of GLD-1-mRNAs complexes in Caenorhabditis nematodes — Evolutionary dynamics of GLD-1-mRNAs complexes in Caenorhabditis nematodes — Evolutionary Dynamics of GLD-1–mRNA Complexes in Caenorhabditis Nematodes — Supplementary Data 

# Evolutionary Dynamics of GLD-1–mRNA Complexes in *Caenorhabditis* Nematodes

## Supplementary Data

files

**Files in this Data Supplement:**

- Supplementary Data - zip file
