# Supplementary material for: Evolutionary Dynamics of GLD-1–mRNA Complexes in Caenorhabditis Nematodes
Source: Genome Biol Evol. 2014 Dec 9;7(1):314–35. doi: 10.1093/gbe/evu272 (PMC4316625; doi:10.1093/gbe/evu272)
Supplement: Supplementary Data [file supp_evu272_suppl_data.zip › Supplemental Information_v4.3.doc]

**SUPPLEMENTAL MATERIALS**

**List of Supplementary Tables:**

**Table S1:** list of proteins included in the alignment and phylogeny (1 sheet)

**Table S2:** the 965 Cbr-GLD-1-associated transcripts identified in this study (1 sheet)

**Table S3:** significant GO terms of Cbr-GLD-1-associated transcripts (based on their *C. elegans* homologs) (2 sheets)

**Table S4**: GBM_finder results on our 965 Cbr-GLD-1 target "UTR sequences" (3 sheets)

**Table S5:** List of the 22 motifs found by the 5 motif discovery programs that were inputted to STAMP AND the identity of STAMP's best match to the RBPDB (and other characteristics) for the 16 motifs for which it detected a convincing match (2 sheets)

**Table S6:** taxonomic breakdown of GLD-1 targets conserved in other species (5 sheets)

**Table S7:** the *C. briggsae*-specific Cbr-GLD-1 targets (1 sheet)

**Table S8:** overlap lists and Venn diagrams regarding the statistical analysis of the Wright et al. data. The first two sheets describe their version of "real vs. mock" expression comparisons and the genes in common between their own Fold Change Ce-GLD-1 target list and our analysis of their "real vs. mock" measurements with SAM and EDGE. The last two tabs describe the overlap of their "real vs. mock" expression comparisons with their "real vs. total" expression comparisons [as we did for our own *C. briggsae* data] (4 sheets)

**Table S9:** the overlap between our 965 *C. briggsae* GLD-1-associated genes and the top 965 Cbr Fold Change genes as calculated from our data (1 sheet)

**Table S10:** the identity of GLD-1 target genes IN COMMON between Ce- and Cbr-GLD-1 (2 sheets)

**Table S11:** GLD-1 targets found ONLY in Beadell et al., ONLY in Jungkamp et al., ONLY in Wright et al., and ONLY on Wright+Jungkamp (4 sheets)

**Table S12:** correlations between SAM enrichment scores/fold changes and the GBM scores of Wright et al. (3 sheets)

**Table S13:** GLD-1 binding motif position and identity among the 97 pairs of putative orthologs robustly associated with GLD-1 in both *C. elegans* and *C. briggsae* (8 sheets)

**Supplementary Text and Figures**

*Chicken anti-GLD-1 antibody recognizes native GLD-1*

In adults, GLD-1 in *C. elegans* is expressed only in the distal gonad, with staining initiating within the germline stem cell "mitotic zone," increasing in intensity as germ cells enter meiosis, and then tapering as cells progress further through prophase I. Native *C. briggsae* GLD-1 is recognized by the chicken anti-GLD-1 antibody developed for this work. As described in Nayak et al. (2005), *C. briggsae* GLD-1's adult expression pattern is the same as in *C. elegans*. We largely followed protocols for dissecting gonads, fixation, and antibody/DAPI staining from the laboratory of Dr. Tim Schedl (Washington University in St. Louis School of Medicine), using the methanol/formaldehyde fix for 10-15 minutes. Chicken anti-GLD-1 was used at 1:4000.

**Figure S1. Chicken anti-GLD-1 antibody recognizes native GLD-1.** Fixed, dissected gonad of a *C. briggsae* wild-type hermaphrodite shown (A) with differential interference microscopy (DIC), (B) Hoechst-stained to observe DNA morphology, and (C) stained with chicken anti-GLD-1. The Cbr-GLD-1 pattern here is the same as observed by Nayak

*Intensity-dependent bias and non-normality of RIP-chip data*

See main text for discussion.

**Figure S2. Intensity-dependent bias and non-normality of RIP-chip data.** Top: Intensity ratio (M) vs. average intensity (A) plot of log_2_-transformed anti-GLD-1 IP vs. anti-IgY (left) and total input mRNA (right) intensity values from representative biological replicates, respectively. Bottom: Quantile-Quantile (Q-Q) plots of log_2_-transformed anti-GLD-1 IP mRNA vs. anti-IgY mRNA (left) and anti-GLD-1 IP mRNA vs. total input mRNA (right) intensity value quantiles vs. normal theoretical quantiles.

*Normalization of Cbr-GLD-1 RIP microarray data*

See main text for discussion.

**Figure S3. Normalization of Cbr-GLD-1 RIP microarray data.** Top: 2x2 matrix of data manipulations tested for both anti-GLD-1 IP vs. mock anti-IgY IP and anti-GLD-1 IP vs. total input mRNA expression comparisons: background correction (either none or *norm-exp* as implemented in *limma* (Silver et al. 2009)) plus normalization (either median scaling or eCADS (Dabney and Storey 2007)). Bottom: Individual array boxplots of (A) *normexp* background corrected, eCADS normalized intensities for five biological replicates of anti-GLD-1 IP and anti-IgY IP microarray intensities, and (B) un- background-corrected, eCADS normalized intensities for three biological replicates of anti-GLD-1 IP and total mRNA microarray intensities.

*SAM d-statistic scores versus fold change measurements for significant probes*

See main text for discussion.

**Figure S4. SAM d-statistic scores versus fold change measurements for significant probes.** Top: SAM d-statistic vs. fold change measurement for individual probes from the anti-GLD-1 vs. mock IP expression comparison significant at the level of FDR=~0. Bottom: SAM d-statistic vs. fold change measurement for individual probes from the anti-GLD-1 vs. total input mRNA expression comparison significant at the level of FDR <2%. In both panels, fold change measurements were calculated by SAM.

*Predicting transcript association with* GLD-1

Wright et al. (2011) created a script, *GBM_finder*, to identify GLD-1-binding motifs (GBMs) present in input sequences and to assign enrichment scores to motifs based on quantification of their RIP-chip *C. elegans* GLD-1 target data. We used the *GBM_finder* to assess whether *C. elegans* GBMs and their associated enrichment scores could explain the IP enrichment of *C. briggsae* GLD-1 mRNA targets. For the UTR-like sequences we obtained for the 965 Cbr-GLD-1-associated transcripts identified here (see *Methods*), the correlation of SAM statistics with *GBM_finder* motif scores was only r=0.14. This value increased to r=0.31 when we used fold change values to determine Cbr-GLD-1 targets, as in Wright et al. (2011), instead of SAM *d-score* statistics as a measure of enrichment (Table S13).

We further probed the sensitivity of GBM enrichment scores to the method of quantifying differential gene expression by reanalyzing the two *C. elegans* anti-GLD-1 IP vs. mock IP data sets of Wright et al. (2011) (but not the anti-GLD-1 IP vs. input RNA data set, as this comparison was not used to create *GBM_finder* in that work) with SAM and EDGE. This yielded 980 statistically significant Ce-GLD-1 targets in common between the two programs (using no more than FDR of 10% for any expression comparison), and this list is 79.1% identical to the original Wright et al. (2011) 948 fold change targets. However, using the full-length transcript of each gene product (WormBase release WS220) only produced a correlation of r=0.17 between SAM scores and GBM scores; this correlation does not improve if we use only the 3'UTRs of these 980 statistically enriched genes rather than their full transcripts (Table S13). Thus, *GBM­finder* predictions may not accurately predict the degree of *C. briggsae* GLD-1 association with its targets, or the computation may be sensitive to the use of exact UTR sequences, which we do not yet have in *C. briggsae*.

*Elaboration of* GLD-1 *binding motif (GBM) conservation among putatively orthologous gene pairs possessing 5’UTR GBMs*

We find that 32/84 (38%) of putatively orthologous gene pairs contain a member with a GBM of score greater than “level 0” in its 5’UTR/upstream sequence, a measure of significant GLD-1 association (Wright et al. 2011). 10 of these gene pairs contain only 5’UTR GBMs in the C. elegans version, 5 contain only GBMs in the “5’UTR” in the C. briggsae version (though an additional 6 pairs might be included here but are not because of very small 5’UTRs (<5bp) in their C. elegans counterpart), and 17 pairs contain 5’UTR GBMs in both species (including 5 gene pairs in which one of the orthologs contains only 0-score GBMs) (Table S13).

Of the 17 orthologous pairs with GBMs in both genes’ 5’UTRs, only a single pair did not also possess obvious conservation in its 3’UTR, rnr-2. (Another two genes possess 5’UTR GBM conservation, but ambiguous 3’UTR GBM conservation, nasp-2 and C05C10.5\CBG09062.) Of the remaining 14 gene pairs, 13 have detectable 3’UTR GBM site conservation. Interestingly, though it has GBMs in all of its 5’ and 3’UTR sequences, we find that the R02F2.1\CBG05210 gene pair displays no site conservation in either UTR.

Of the 10 pairs of genes that possess 5’UTR GBMs only in C. elegans for which all UTR sequences are available, three have detectable conservation of 3’UTR GBMs as well, asd-1, hum-2 and ¬rme-2, and one pair maintains ambiguous GBM 3’UTR conservation, ttr-53. The remaining 7 pairs of orthologs, him-1, ile-1, lst-1, ntp-1, tsp-12, and K07H8.2\CBG01586, all seem to have independent or rapidly evolving GBMs in their 3’ UTRs.

Of the 5 pairs of orthologs that possess 5’UTR GBMs only in C. briggsae for which all UTR sequences are available, 3 show either rapid or independent evolution of their 3’UTR GLD-1 binding sites, ima-2, puf-11, C48B6.10/CBG12731, whereas one pair, rskn-1, displays ambiguous GBM conservation in its 3’UTR.
